# Impact of cricothyroid membrane puncture anesthesia on elderly patients undergoing endotracheal intubation after anesthesia induction: a clinical study

**DOI:** 10.3389/fphys.2025.1600369

**Published:** 2026-03-11

**Authors:** Yanbing Wang, Hongchuan Zhao, Yanjiao Liang, Xiaoli Li, Xiao Bi, Hao Lian

**Affiliations:** 1 Department of Anesthesiology, Chifeng Cancer Hospital, Chifeng, China; 2 Department of Surgical Oncology, Chifeng Cancer Hospital, Chifeng, China

**Keywords:** endotracheal intubation, cricothyroid membrane puncture, surface anesthesia, hemodynamics, recovery period, elderly patient

## Abstract

**Objective:**

This study investigated the clinical effects of cricothyroid membrane puncture (CMP) anesthesia surface anesthesia following anesthesia induction on elderly patients undergoing endotracheal intubation (ETI).

**Methods:**

Eighty elderly patients scheduled for general anesthesia with endotracheal intubation at our hospital from January to December 2023 were enrolled and randomly assigned (n = 40 each) to a study group or a control group. After intravenous anesthesia induction, the study group received 2% lidocaine via CMP for surface anesthesia, while the control group received no CMP administration. The incidence of coughing during extubation in the emergence phase, first-attempt intubation success, hypoxemia, hypotension, and vasoactive drug use were recorded. Hemodynamic parameters and adverse events were compared between groups.

**Results:**

All patients achieved successful first-attempt intubation. There were no significant differences in hypoxemia incidence between groups (*P* > 0.05). The study group had lower rates of hypotension and vasoactive drug use compared with the control group (*P* < 0.05), as well as a lower incidence of coughing during extubation (*P* < 0.05). At T0, heart rate (HR), systolic blood pressure (SBP), mean arterial pressure (MAP), and SpO_2_ did not differ significantly (*P* > 0.05). At T1-T3, HR, SBP, and MAP were significantly lower in the study group (*P* < 0.05), with no significant differences in SpO_2_ (*P* > 0.05). Total adverse event rates were similar between groups (*P* > 0.05).

**Conclusion:**

CMP surface anesthesia following anesthesia induction in elderly patients provides favorable clinical effects by improving hemodynamic stability and reducing coughing during emergence, without increasing adverse events.

## Introduction

Difficult airway management during anesthesia remains a major contributor to perioperative morbidity and mortality, with potential preventable consequences such as airway trauma, hypoxic brain injury, and even death (Joffe et al., 2019). Many patients requiring endotracheal intubation (ETI) present with borderline respiratory and circulatory reserve, hemodynamic instability, and limited oxygen stores. These factors constrain the dosage of general anesthetics and the allowable duration for intubation, making ETI particularly high-risk in this population. In elderly patients, age-related degenerative changes in the cardiovascular system reduce their tolerance to hemodynamic fluctuations, while altered pharmacokinetics influence drug distribution, half-life, and clearance (Uysa et al., 2012). Consequently, controlling induction and maintenance of general anesthesia is challenging, with increased susceptibility to severe blood pressure fluctuations, peri-induction complications, respiratory depression, and delayed emergence due to incomplete postoperative drug metabolism.

Clinically, for patients with predicted difficult airways, anesthesiologists often adopt a slow-induction strategy with amnesic analgesia while preserving spontaneous respiration. In such cases, surface anesthesia with local anesthetics via cricothyroid membrane puncture (CMP) is widely employed (Andruszkiewicz et al., 2010). CMP anesthesia can effectively attenuate airway reflexes and blunt hemodynamic surges caused by tracheal intubation, thereby improving ETI safety in difficult airway scenarios (Chen et al., 2018). Anatomically, the cricothyroid membrane is the thinnest portion between the laryngeal cavity and trachea, with a superficial location and absence of major vessels, nerves, and rigid structures, making it a reliable and safe access point for percutaneous subglottic airway interventions (Kristensen et al., 2016). However, most CMP anesthesia procedures are performed in awake patients, and there is limited evidence on its application after anesthesia induction in elderly individuals. The potential benefits of combining CMP surface anesthesia with ETI under deep sedation—particularly in improving peri-induction hemodynamic stability and reducing emergence-phase airway complications—have not been fully elucidated. This study aimed to evaluate the clinical effects and safety of CMP anesthesia followed by ETI in elderly patients after induction, thereby addressing a current evidence gap and offering insights into optimizing airway management in high-risk populations.

## Materials and methods

### Ethical statement

The study was reviewed and approved by the ethics committee of Chifeng Cancer Hospital, and all patients signed an informed consent form.

### Study subjects

Eighty elderly patients scheduled for general anesthesia requiring ETI in Chifeng Cancer Hospital during the period of January 2023-December 2023 were selected as the study subjects. Using a random number table, participants were assigned to a study group (CMP surface anesthesia after induction) or a control group (no CMP surface anesthesia after induction), with 40 cases each.

Inclusion criteria: Patients aged 65–75 years; Patients with American Society of Anesthesiologists (ASA) physical status I-II (Sankar et al., 2014); Mallampati classification Ⅰ-Ⅲ (Stutz and Rondeau, 2025).

Exclusion criteria: Patients with upper respiratory tract infection within the previous week; Patients who died during ETI; Patients with allergy to study medications; Patients with essential hypertension; Patients with psychiatric disorders.

### Sample size calculation

The required sample size was estimated using a statistical power-based approach with G*Power software version 3.1.9.7 (University of Düsseldorf, Germany). Parameters were set as follows: power (1–β) = 0.80, α = 0.05, effect size = 0.70, and number of groups = 2. The calculation indicated that a minimum of 68 participants was needed. Considering an anticipated attrition rate of 15%, the sample size was rounded up, resulting in a final enrollment target of 80 participants.

### Randomization and blinding

Participants were randomly assigned to groups using a random number table. The 80 eligible patients were sequentially numbered from 1 to 80 according to enrollment order. Random numbers were drawn from the table, sorted in ascending order, and the first 40 numbers were allocated to the control group, while the remaining 40 were allocated to the study group. The randomization sequence was prepared and sealed by an independent statistician, and opened only immediately prior to intervention. Due to the nature of the intervention, a single-blind design was adopted, with participants unaware of group allocation. Operator blinding was not feasible. To minimize detection bias, outcome assessments were performed by independent evaluators blinded to the intervention, all of whom underwent standardized training prior to the study.

### Anesthesia induction and intervention

All patients underwent standard monitoring upon entering the operating room, followed by general anesthesia induction. Midazolam 0.03 mg/kg and sufentanil 0.05 μg/kg were administered, and invasive arterial blood pressure monitoring was established. Sequential induction was then performed with etomidate 2 mg/kg, rocuronium 1 mg/kg, and sufentanil 0.25 μg/kg, with identical drug administration order for all patients. Five minutes after intravenous induction, patients in the study group received CMP surface anesthesia. Patients were placed in the supine position with maximal head extension to fully expose the anterior neck. The larynx was stabilized with the middle finger and thumb of the left hand, and the puncture site was identified at the center of the cricothyroid space. After donning sterile gloves, disinfecting with povidone-iodine, and placing a sterile drape, the operator fixed the cricothyroid membrane with the left thumb and index finger, and advanced a puncture needle held in the right hand perpendicular to the skin and directed caudally. The needle was advanced approximately 1 cm until loss of resistance was felt, and free air was aspirated smoothly, confirming entry into the tracheal lumen. The needle and syringe were then stabilized with the left hand while the right hand rotated the needle gently and injected 5 mL of lidocaine solution at a steady rate. After injection, the needle was withdrawn, and patients were encouraged to take deep breaths and cough. In the control group, no CMP anesthesia was performed after induction. Drug dosages were adjusted based on the patient’s level of consciousness, hemodynamic responses, and intubation conditions. Once loss of consciousness was achieved, endotracheal intubation was performed with a standard endotracheal tube.

### Observation indicators

Clinical data such as age, gender, body mass index, and ASA classification were collected from all study subjects.

The primary outcomes were the incidence and severity of coughing during extubation in the emergence phase, assessed on a 0–3 scale (Chen et al., 2010). 0 = no cough or breath-holding, patent spontaneous or mask ventilation; 1 = mild cough or breath-holding ≤5 s, no obvious head movement, smooth mask ventilation, no change in SpO_2_; 2 = moderate cough or breath-holding >5 s and <15 s, noticeable head shaking, some resistance to mask ventilation but basic ventilation maintained, SpO_2_ < 92%; 3 = severe cough with head lift from the bed or inability to maintain effective ventilation with mask pressure, SpO_2_ < 92%. Coughing rates during extubation, first-attempt intubation success, incidence of hypoxemia (SpO_2_ < 90% for >60 s), hypotension (mean arterial pressure (MAP) change >30%), and vasoactive drug use were recorded for both groups.

Secondary outcomes included heart rate (HR), systolic blood pressure (SBP), MAP, and SpO_2_ at four time points: before induction (T0), during intubation (T1), 1 min after intubation (T2), and 5 min after intubation (T3). Adverse events related to CMP anesthesia—such as local anesthetic allergy, cardiovascular reactions, puncture site bleeding, and oropharyngeal pain—were documented at postoperative 24 h and 1 week follow-up.

### Statistical analysis

SPSS 21.0 software (IBM, N.Y, United States) and GraphPad Prism 6.01 software (Graph Pad Inc., CA, United States) were utilized for data analysis. Normality of continuous variables was assessed using the Shapiro-Wilk test. Data conforming to a normal distribution were expressed as mean ± standard deviation (SD), and between-group comparisons were performed using the independent-samples t-test. Categorical variables were expressed as percentages (%) and compared using the χ^2^ test or Fisher’s exact test, as appropriate. Hemodynamic parameters at different time points were analyzed using repeated-measures analysis of variance (ANOVA), with pairwise comparisons within groups adjusted by the Bonferroni correction. No missing data were present in the final dataset; therefore, no additional imputation was performed. *P* < 0.05 was considered statistically significant.

## Results

### Clinical data

No significant differences were observed between the two groups in age, gender, body mass index, ASA grading, and Mallampati classification between the two groups found no differences (*P* > 0.05) (Table 1).

**TABLE 1:** Comparison of clinical data between the two groups.

Indicators		Control group (n = 40)	Study group (n = 40)	*χ* ^*2*^ */t* value	*P* value
Age (years)		70.25 ± 2.69	70.38 ± 2.89	0.200	0.842
Gender	Male	23 (57.50%)	21 (52.50%)	0.202	0.653
	Female	17 (42.50%)	19 (47.50%)		
Body mass index (kg/m^2^)		26.31 ± 2.59	25.76 ± 2.96	0.889	0.377
ASA grading	Ⅰ	13 (32.50%)	15 (37.50%)	0.220	0.639
	II	27 (67.50%)	25 (62.50%)		
Mallampati classification	I	14 (35.00)	15 (37.50)	0.020	0.888
	II	16 (40.00)	13 (32.50)		
	III	10 (25.00)	12 (30.00)		

Note: ASA, american society of anesthesiologists.

### Clinical outcomes

All patients in both groups achieved successful intubation on the first attempt. The incidence of hypoxemia did not differ significantly between the two groups (*P* > 0.05). However, the study group exhibited markedly lower rates of hypotension and vasoactive drug use compared with the control group (*P* < 0.05). These findings suggest that performing ETI following CMP surface anesthesia provides favorable clinical outcomes in elderly patients (Table 2).

**TABLE 2:** Comparison of clinical outcomes between the two groups.

Indicators	Control group (n = 40)	Study group (n = 40)	*χ* ^*2*^ value	*P* value
Incidence of hypoxia	2 (5.00%)	1 (2.50%)	0.346	0.556
Incidence of hypotension	6 (15.00%)	1 (2.50%)	3.914	0.048
Utilization rate of vasoactive drugs	13 (32.50%)	5 (12.50%)	4.588	0.032

### Choking cough during the recovery period

The choking rate during the recovery period in the study group was lower than that in the control group (*P* < 0.05). The results show that CMP anesthesia followed by ETI is beneficial in reducing the incidence of choking during the recovery period in elderly patients (Table 3).

**TABLE 3:** Comparison of the occurrence of choking cough during the recovery period between the two groups.

Indicators	Control group (n = 40)	Study group (n = 40)	*χ* ^*2*^ value	*P* value
0 points	34 (85.00%)	39 (97.50%)	—	—
1 point	3 (7.50%)	1 (2.50%)	—	—
2 points	3 (7.50%)	0 (0.00%)	—	—
3 points	0 (0.00%)	0 (0.00%)	—	—
Choking rate during extubation	6 (15.00%)	1 (2.50%)	3.914	0.048

### Hemodynamic conditions

At T0, the comparison of HR, SBP, MAP and SpO_2_ between the two groups was not statistically significant (*P* > 0.05). At T1-T3, HR, SBP, and MAP were significantly lower in the study group (*P* < 0.05), with no significant differences in SpO_2_ (*P* > 0.05). The results unveil that CMP anesthesia followed by ETI is beneficial in improving hemodynamic indices in elderly patients (Table 4).

**TABLE 4:** Comparison of hemodynamic conditions between the two groups.

Indicators		Control group (n = 40)	Study group (n = 40)	*t* value	*P* value
HR (beats/min)	T_0_	90.18 ± 7.45	90.88 ± 8.12	0.402	0.689
	T_1_	120.88 ± 7.09*	106.85 ± 7.85*	8.384	<0.001
	T_2_	106.95 ± 6.30*	99.65 ± 6.52*	5.095	<0.001
	T_3_	94.93 ± 7.38*	83.70 ± 5.39*	7.771	<0.001
SBP (mmHg)	T_0_	121.15 ± 6.61	120.45 ± 8.11	0.423	0.673
	T_1_	116.85 ± 8.23*	107.68 ± 7.91*	5.082	<0.001
	T_2_	121.45 ± 8.54	116.33 ± 8.09*	2.755	0.007
	T_3_	114.08 ± 13.05*	108.90 ± 7.07*	2.206	0.030
MAP (mmHg)	T_0_	82.38 ± 4.76	81.93 ± 4.07	0.454	0.651
	T_1_	80.55 ± 3.03*	76.80 ± 3.65*	5.003	<0.001
	T_2_	78.03 ± 3.91*	74.08 ± 3.65*	4.666	<0.001
	T_3_	76.68 ± 4.36*	72.95 ± 3.31*	4.304	<0.001
SpO_2_ (%)	T_0_	92.45 ± 1.58	92.18 ± 1.71	0.747	0.458
	T_1_	96.40 ± 2.12*	96.63 ± 2.48*	0.436	0.664
	T_2_	97.78 ± 1.87*	98.35 ± 1.19*	1.639	0.105
	T_3_	98.23 ± 2.50*	98.90 ± 1.77*	1.396	0.167

Note: HR, heart rate; SBP, systolic blood pressure; MAP, mean arterial pressure; SpO_2,_ blood oxygen saturation; * indicates *P* < 0.05 compared with T0.

### Occurrence of adverse reactions

No cases of local anesthetic allergy occurred in either group. In the control group, one patient experienced a transient cardiovascular reaction (brief arrhythmia), which resolved spontaneously within several minutes. Both groups had two cases of puncture site bleeding, all of which were controlled by applying sterile gauze compression for 5 min without hematoma formation or interference with subsequent procedures. No cases of oropharyngeal pain occurred in the control group, whereas two cases of mild oropharyngeal pain (VAS score 2–3) were reported in the study group, all resolving spontaneously within 24 h postoperatively. The overall incidence of adverse events was 7.50% in the control group and 10.00% in the study group, with no statistically significant difference between groups (*P* > 0.05). These findings indicate that ETI following CMP surface anesthesia is well tolerated and demonstrates a favorable safety profile in elderly patients (Table 5).

**TABLE 5:** Comparison of the occurrence of adverse reactions in the two groups.

Indicators	Control group (n = 40)	Study group (n = 40)	*χ* ^*2*^ value	*P* value
Allergy to local anesthetic	0 (0.00%)	0 (0.00%)	—	—
Cardiovascular response	1 (2.50%)	0 (0.00%)	—	—
Bleeding at the puncture site	2 (5.00%)	2 (5.00%)	—	—
Oropharyngeal pain	0 (0.00%)	2 (5.00%)	—	—
Total incidence rate	3 (7.50%)	4 (10.00%)	0.157	0.692

## Discussion

During ETI, induction agents such as midazolam, propofol, etomidate, and fentanyl are commonly used (Allen et al., 2023). Most cases are induced while retaining spontaneous breathing; however, even with appropriate anesthetic dosing, elderly patients often experience hypotension after induction due to frailty and comorbidities (Sudfeld et al., 2017). Large hemodynamic fluctuations during intubation further increase perioperative risk (Ranjbar et al., 2018). Previous studies have shown that CMP anesthesia during awake fiberoptic intubation yields superior intubation conditions, shorter intubation times, and higher patient and operator satisfaction than conventional topical anesthesia (Wang et al., 2021). Moreover, CMP injection provides more effective airway anesthesia than nebulized lidocaine during diagnostic bronchoscopy (Isaac et al., 1990). Building on these findings, we applied CMP surface anesthesia after anesthesia induction, a less-reported approach, to explore its value in elderly patients.

In this study, both groups achieved a 100% first-attempt intubation success rate, confirming that CMP anesthesia does not compromise technical feasibility even in elderly patients. Notably, the CMP group had lower rates of hypotension and reduced vasoactive drug use, indicating better peri-intubation hemodynamic stability. This aligns with the pharmacological profile of lidocaine—rapid onset, wide dispersion, strong mucosal permeability, and the ability to block peripheral cough receptors and vagal reflexes, relax airway smooth muscle, and suppress arrhythmias (Rahimi et al., 2018). Modern studies also show that lidocaine can attenuate airway hyperreactivity induced by kinins, reduce forced expiratory volume decline, and blunt cardiovascular responses to airway manipulation (Sun et al., 2021). While some studies report no significant association between lidocaine use and hypotension or heart rate changes (Kim et al., 2025), our findings suggest that CMP delivery may enhance its hemodynamic benefits by ensuring rapid, concentrated mucosal exposure.

Compared with other surface anesthesia methods, CMP offers the advantage of delivering a larger volume of lidocaine directly to the subglottic area in a short time, resulting in faster onset and more complete airway anesthesia. This targeted delivery may explain its superiority over nebulized or sprayed lidocaine in suppressing airway reflexes and stabilizing hemodynamics. Clinically, this could be particularly advantageous in elderly patients with limited tolerance for sympathetic surges during ETI.

The absence of significant differences in adverse reactions (e.g., local anesthetic allergy, bleeding, or oropharyngeal pain) between groups further supports the safety profile of CMP anesthesia. Topically applied agents with analgesic properties primarily act on peripheral pain pathways, thereby reducing systemic absorption and minimizing the risk of widespread adverse effects (Gudin and Nalamachu, 2020). Lidocaine is a local anesthetic, which can block the Na^+^ channel and produce local anesthetic effect (Hermanns et al., 2019). In the CMP anesthesia approach, a relatively large amount of lidocaine comes into direct contact with the airway surface within a short time, resulting in rapid anesthetic onset. The cricothyroid administration route has been shown to be both safe and effective for topical anesthesia during endobronchial ultrasound-guided transbronchial needle aspiration (Mittal et al., 2021). Moreover, at low concentrations, lidocaine poses minimal toxicity risk while maintaining respiratory and hemodynamic stability (Liu et al., 2020). Yang et al. reported that the low incidence of throat pain associated with lidocaine use may be attributable to its stabilizing effects on both central and peripheral nervous systems, further supporting its potential role in mitigating anesthesia-related adverse effects (Han et al., 2024).

In this study, the increases in HR, SBP, and MAP in the observation group were lower than those in the control group at T1, T2, and T3, indicating that CMP anesthesia contributed to greater circulatory stability and more effective attenuation of intubation-related irritation. This finding is clinically relevant, as airway manipulation often triggers pronounced sympathetic reflex activation, leading to significant hemodynamic changes. With reduced vagal stimulation during ETI, the incidence of tachycardia rises correspondingly (Mendonca et al., 2022). Such hemodynamic instability can diminish cardiac output, impair tissue perfusion and oxygen delivery, and promote anaerobic metabolism, resulting in lactic acidosis, progressive organ dysfunction, and potentially death (Kumar et al., 2021). Therefore, achieving adequate airway surface anesthesia before ETI is crucial for minimizing airway stimulation, preventing cardiovascular adverse reactions during intubation, and maintaining hemodynamic stability (Tung et al., 2020).

Choking during emergence from anesthesia is a common and potentially dangerous event that can induce laryngospasm, hypoxia, or hypercapnia (Tang et al., 2023). Previous studies have demonstrated that intravenous administration of lidocaine during surgery can reduce respiratory complications such as coughing (Yang et al., 2020). Similarly, administering lidocaine intravenously before anesthesia in premedicated dogs effectively alleviates the coughing response associated with ETI (Panti et al., 2016). In line with these observations, our study found that CMP anesthesia with lidocaine significantly reduced the incidence of choking cough during the recovery period in elderly patients, thereby enhancing airway safety and patient comfort.

This study has several limitations. First, the sample size was relatively small (n = 80) and conducted at a single center; further subgroup analyses would markedly reduce statistical power, potentially affecting the stability and reliability of the findings. Future multi-center studies with larger and more diverse patient populations are needed to enhance external validity and allow for more precise subgroup analyses. Second, due to the invasiveness of CMP and the specific drug administration method, a single-blind design was used, and blinding of operators was not feasible. Although standardized scoring scales and independent, trained evaluators were employed to minimize bias in subjective outcomes such as the cough score, the influence of assessor experience cannot be completely excluded. Third, patients with essential hypertension were not included, which may limit the generalizability of the results to this population. Finally, the study focused on the immediate safety of tracheal intubation after CMP, with only short-term adverse events recorded; future research should incorporate longer follow-up periods to assess long-term safety and outcomes.

In conclusion, this study provides evidence that CMP anesthesia, when performed after induction in elderly patients, enhances the safety of ETI by stabilizing hemodynamics and reducing recovery-related adverse events. Given the high perioperative risks in elderly individuals—such as age-related cardiovascular changes, reduced physiological reserve, and multiple comorbidities—our findings offer a practical and targeted strategy to optimize airway management in this population. By attenuating the sympathetic response to tracheal tube stimulation, CMP anesthesia helps to minimize hemodynamic fluctuations and reduce the incidence of choking during emergence, ultimately contributing to improved perioperative stability and patient outcomes.

To our knowledge, this is the first controlled clinical study to evaluate CMP anesthesia performed after induction—rather than in the traditional awake setting—in elderly surgical patients requiring ETI. The results demonstrate that this approach not only maintains procedural safety but also confers measurable benefits in hemodynamic stability and recovery quality without increasing overall adverse event rates. This expands the potential application of CMP anesthesia from difficult airway cases to routine airway management in high-risk elderly patients, offering a new option that is both feasible and effective in the intraoperative setting. Anesthesiologists can consider incorporating CMP anesthesia after induction into the airway management protocol for elderly patients at risk of peri-intubation hemodynamic instability, particularly those with limited tolerance for blood pressure fluctuations or prone to severe choking during emergence. Successful implementation requires standardized operator training, strict adherence to aseptic technique, careful patient selection, and close perioperative monitoring. Integrating this method into routine practice could enhance patient safety, improve comfort during recovery, and potentially reduce the need for vasoactive support in elderly surgical populations.

## Data Availability

The original contributions presented in the study are included in the article/supplementary material, further inquiries can be directed to the corresponding author.
